# A mini-review on co-supplementation of probiotics and medicinal herbs: Application in aquaculture

**DOI:** 10.3389/fvets.2022.869564

**Published:** 2022-11-02

**Authors:** Lee Seong Wei, Khang Wen Goh, Noor Khalidah Abdul Hamid, Zulhisyam Abdul Kari, Wendy Wee, Hien Van Doan

**Affiliations:** ^1^Department of Agricultural Sciences, Faculty of Agro-Based Industry, Universiti Malaysia Kelantan, Jeli, Kelantan, Malaysia; ^2^Advanced Livestock and Aquaculture Research Group, Faculty of Agro-Based Industry, Universiti Malaysia Kelantan, Jeli, Kelantan, Malaysia; ^3^Faculty of Data Science and Information Technology, INTI International University, Nilai, Malaysia; ^4^School of Biological Sciences, Universiti Sains Malaysia, Pulau Pinang, Malaysia; ^5^Center of Fundamental and Continuing Education, Universiti Malaysia Terengganu, Kuala Terengganu, Malaysia; ^6^Department of Animal and Aquatic Sciences, Faculty of Agriculture, Chiang Mai University, Chiang Mai, Thailand; ^7^Science and Technology Research Institute, Chiang Mai University, Chiang Mai, Thailand

**Keywords:** aquaculture, medicinal herb, immune system, disease resistance, probiotic, synergy, growth performance

## Abstract

The aquaculture industry is geared toward intensification and successfully meets half of the world's demand for fish protein. The intensive farming system exposes the animal to the risk of disease outbreaks, which has economic consequences. Antibiotics are commonly used for the health management of aquaculture species. However, this has several drawbacks, including the increase in antibiotic resistance in pathogenic bacteria and the entry of antibiotic residues into the human food chain, which is a public health and environmental concern. The potential of probiotics, prebiotics, synbiotics, and medicinal herbs as alternatives to antibiotics for the health management of aquaculture species has been investigated in numerous studies. This review discusses the potential use of combinations of probiotics and medicinal herbs as prophylactic agents in aquaculture, along with the definitions, sources, and modes of action. The positive aspects of combining probiotics and medicinal herbs on growth performance, the immune system, and disease resistance of aquaculture species are also highlighted. Overall, this review addresses the potential of combinations of probiotics and medicinal herbs as feed additives for aquaculture species and the key role of these feed additives in improving the welfare of aquaculture species.

## Introduction

Aquafeeds and aquaculture health management could be described as a critical sector for the sustainability of the aquaculture industry. Aquaculture contributed 48% of total global fish production in 2019 and, in spite of the pandemic (SARS-CoV-2 virus), remains the fastest growing industry, according to latest FAO report (1). High consumer market demand and advanced technologies have led the industry to drastically shift to more intensive farming systems to increase yields, which threatens the health of farmed fish through stress. Synthetic antibiotics are used in aquaculture because of their affordable price, accessibility, and efficacy to immediately control disease outbreaks (2). However, the misuse of synthetic antibiotics in aquaculture has led to an increase in antibiotic-resistant cases in pathogenic bacteria in aquaculture systems. Some reports also show that antibiotic residues enter the human food chain (3). Therefore, a different approach should be applied using non-toxic substances safer for the health management of aquaculture species, such as effective microbes and herbs (4). A survey has shown that shrimp farms in China, Thailand, and Vietnam have switched to feed additives, such as medicinal herbs, probiotics, and prebiotics, as alternatives to antibiotics for disease prevention (5).

Probiotics are live microorganisms that, when administered in adequate amounts, confer a health benefit on the host (6). Probiotics are widely used to prevent and treat various diseases in public health (7) and aquatic animals (8) by improving their immune response (9, 10). Lactic acid bacteria (LAB) are the most important probiotic group used in humans and animals (11) with the ability to ferment glucose into lactic acid, acetic acid, and ethanol. This probiotic group occurs naturally in dairy products and plant materials. For a long time, LAB has been used in the food and beverage industry to make dairy products, such as cheese and yogurt. In aquaculture, LAB have been used to promote the growth of the gut microbiota and counteract fish pathogens, such as *Streptococcus iniae* and *Lactococcus garvieae* (12). To date, there have been several recent studies using probiotics in aquaculture, namely *Bacillus subtilis* (13), *Lactococcus lactis L19* (14), *Lactobacillus plantarum* (15), and *Lactococcus lactis subsp lactis PTCC 1403* (16).

Medicinal herbs have been an essential part of ancient medicine, such as Chinese medicine, Unani medicine, and Ayurveda, for centuries and are gaining popularity worldwide as an alternative medicine to maintain wellbeing and treat ailments, diseases, and health conditions in humans (17). Many studies have shown that plant products can be used as feed additives in aquaculture, as well to promote growth, strengthen the immune system, and stimulate disease resistance in aquaculture species (Table 1). Though the preparation of medicinal herbs is too expensive to be used widely for animals, some of the waste from the medicinal herb industry, especially from ethanol or water extraction, still containing 30–50% essential bioactive compounds, can be used as feed additive in animal husbandry, including aquaculture (25). This could help to solve the problem related to accumulation of by-products from the medicinal herb industry worldwide. It is estimated that the medicinal herb industry generates about 30 million tons of waste annually (26).

**Table 1:** Recent studies of medicinal herb application in aquaculture.

**Medicinal herbs**	**Aquaculture species**	**Description**	**References**
Yacon, ginger, blueberry	Olive flounder, *Paralichthys olivaceus*	Stimulate disease resistant to *Streptoccocus iniae*	(18)
Elephant's foot (*Elephantopus scaber*) extract	Nile tilapia, *Oreochromis niloticus*	Better growth performance; Improve immune system; Stimulate disease resistant to *Streptoccocus agalactiae*	(19)
Papaya leaf extract	Red hybrid tilapia	Improve growth performance	(20)
*Hygrophila auriculata*	Fingerling of *Cirrhinus mrigala*	Better growth performance; improve	(21)
*Astragalus memberanaceus, Bupleurum chinense*	White shrimp, *Litopenaeus vannamei*	Better growth performance; improve immune system	(22)
Black garlic	White shrimp, *Litopenaeus vannamei*	Better growth performance; improve immune system; stimulate disease resistant to *Vibrio parahaemolyticus*	(23)
*Bougainvillea glabra leaf*	Nile tilapia, *Oreochromis niloticus*	Better growth performance; increase disease resistant to *Enterococcus faecalis*	(24)

The use of probiotics and medicinal herbs in aquaculture has its costs and benefits. When adding multiple feed additives, there are also conditions where the combination of active ingredients contained in the plant extract and probiotics may not show improvement or may not be beneficial to the fish. However, there are reports showing that the combined use of probiotics and medicinal herbs in aquaculture has been shown to improve fish growth and welfare better than treatment with a single additive (probiotics or herbs) (27–31). Therefore, this mini-review discussed and highlighted the role of medicinal herbs and probiotics in combination for the use in aquaculture.

## Probiotics

Probiotics are considered beneficial and good microorganisms that are usually used to maintain water quality by breaking down ammonia and nitrate in an aquaculture system, and as a feed additive to improve host health (32), increase growth, boost the immune system, and promote disease resistance in aquaculture species (33). Commonly used probiotics include *Aspergillus niger, A*. *oryzae, Candida pintolopesii, Saccharomyces cerevisiae, Bacillus, Bifidobacterium, Enterococcus, Propionibacterium, Pediococcus, Leuconostoc, Streptococcus*, and *Lactobacillus* (34). Probiotics can come from conventional and non-conventional sources (34). Conventional sources of probiotics include dairy products, milk, and stool, while non-conventional sources come from non-dairy fermented foods, non-intestinal sources, and animals' digestive systems (34). Beneficial microorganisms must meet several criteria before they can be recognized as probiotics. These criteria are non-toxicity, inhibition of pathogenic microorganisms, tolerance to acidic environments and bile, and ability to attach to the epithelial cells of the gut (35).

Although the exact mechanism of action of probiotics is unclear, the effect of probiotics on pathogenic microorganisms is associated with several mechanisms, including antimicrobial secretion, competitive adhesion to epithelium and mucosa, reinforcement of the intestinal epithelial barrier, and regulatory effects on the immune system, which are well-known in mammals. In fish, there is evidence of the role of probiotics in regulating intestinal epithelial function by promoting mucus layer formation (36), secretion of antibacterial factors (37), and competitive adhesion to intestinal epithelial cells (38). In general, probiotics are reported to interact with intestinal epithelial cells directly *via* cellular components such as DNA (39), lipoteichoic acids (40), and polysaccharides (41) at the cell surface, as well as indirectly *via* the production of bioactive metabolites (42). Microbe-derived peptides and polysaccharides can activate signaling pathways and alter parameters, such as cytokine release and gut permeability, thus improving the barrier function of the epithelium (43).

Probiotics are widely administered for prophylaxis and public health maintenance. Likewise, probiotics are used in aquaculture health management to prevent disease outbreaks, boost the immune system, and promote the growth of aquaculture species (44). The benefits of using probiotics in aquaculture have been consistently demonstrated. For example, Azad et al. (44) reported the application of commercial probiotics in the diet (Zymetin) and soil (Super PS) of giant freshwater prawn, *Macrobrachium rosenbergii* was the best and cost effective practise for maintaince of water quality and an increase of production. Olmos et al. (45) reported that the use of Bacillus subtilis in aquaculture can improve the growth and disease resistance of shrimp and fish and maintain water parameters in the aquaculture system. In red seabream, *Pagrus major*, the probiotic increases immunological parameters, such as sodium oxide dismutase (SOD) and the alternative complement pathway (ACP) in serum (46). The ACP is involved in direct killing of microorganisms *via* lysis, opsonisation of microorganisms by phagocytosis, chemotactic attraction to the site of inflammation and activation of leukocytes, processing of immune complexes, and induction of specific antibody responses through enhanced localization of antigens on B lymphocytes and antigen-presenting cells. Probiotics, therefore, have great potential for the aquaculture industry.

## Medicinal herbs

Medicinal herbs are botanical therapeutic products derived from plant parts that have been used for 100's of years in alternative medicine and ethnomedicine around the world (47). Depending on the therapeutic elements, the plants' leaves, fruits, seeds, barks, roots, oils, and juices are used as medicine (48). Medicinal herbs contain secondary metabolites or bioactive compounds such as phenolic compounds, terpenoids, and polysaccharides, rich in antioxidants and antimicrobial properties and can treat various health conditions and diseases (17). The preparation of these medicinal herbs involves processes, such as aqueous extraction for therapeutic formulation. The aqueous extraction is a common process for preparing medicinal herbs that produce by-products. These by-products still contain bioactive compounds that could be reused instead of discarded as waste. The remaining bioactive compound available in medicinal herb wastes can be added to animal feed formulation as functional feed additives (49).

Medicinal herbs alone have been shown to strengthen the immune system of aquatic animals. Bioactive compounds such as flavonoids, terpenoids, saponins, and alkaloids in the medicinal herb may provide an alternative to commercial antibiotics as antimicrobial agents in aquaculture (18, 19). Medicinal herbs contain antioxidant properties that can boost the immune system by facilitating nutritional uptake into the gut epithelial cell, and the bioactive plant compound promotes the growth of the gut microbiota (50). However, the use of medicinal herbs in aquafeeds may depend on the dosage, as the plants often contain an anti-nutritive factor and too high a dosage of herbs may not be economical or may have negative effects. For example, the addition of 0.5% garlic improved the growth performance of sterlet sturgeon compared to the control. However, the addition of 1% garlic did not make any improvements in comparison with fish treated with 0.5% garlic (51), indicating that the addition of more garlic extract than the optimal amount of 0.5% to the sterlet sturgeon diet does not improve further fish performance. In red hybrid tilapia, for example, the addition of papaya leaf extract was optimal at 1–2% of the extract, but at a higher level (4%), the growth performance of red hybrid tilapia was negatively affected and did not differ from the control group (20).

## Combined effects of probiotics and medicinal herbs on the growth performance of aquatic animals

Combinations of probiotics and medicinal herbs have been shown to boost growth performance of various aquaculture species, from fish to crustaceans, and from freshwater to marine fish (Table 2) (28, 61). According to the existing literature data, the probiotics used so far in combination with herbal medicines to promote the growth performance of aquaculture species belonged to the group of lactic acid bacteria (LAB), with the exception of the study by Harikrishnan et al. (52) in which the probiotics from the group LAB were combined with medicinal herbs along with yeast, *Saccharomyces cerevisiae*. The medicinal herbs used for the combination with probiotics range from common spices such as shallot (30), peppermint (53), and ginger (54) which also contain various medicinal values, to superfoods such as spirulina (55) and royal oyster mushroom (56). Herbal mixtures commonly used in certain regions were also studied, such as *Allium koreanum, Glycyrrhiza uralensis*, and *Panax ginseng* in Korea (21), *Isatis tinctoria L, Isatis indigodica Fort, Forsythia suspersa Vahl, Corydalis bungeana Turez, Pogostemon cablin* (blanco) Benth, and *Astragalus membranaceus* (Fisch) in China and many others. In addition to mixing the probiotic into the feed containing herb additive, fermentation is also a technique of combining both probiotic and herbs into aquafeed (28, 62, 63).

**Table 2:** Application of probiotic and medicinal herb combination in improving growth performance of aquatic animals.

**Probiotics Dosage**	**Medicinal herbs** **Dosage**	**Fish species**	**Duration**	**References**
Biomin^®^IMBO probiotic 1 and 3%	Persion shallot 1 and 3%	Zebra fish *Danio rerio*	60 days	(30)
*Lactobacillus plantarum, L. acidophilus, L. brevis, Bacillus subtilis*, and *Saccharomyces cerevisiae* Each 0.1% of diet	Combination of *Allium koreanum, Glycyrrhiza uralensis*, and *Panax ginseng* 0.5% of diet	Olive flounder, *Paralichthys olivaceus*	12 weeks	(52)
*Bacillus coagulans* 3,000 CFU/ ml	*Mentha piperita* 2–8 g/ kg diet	Indian carp, *Catla catla*	90 days	(53)
*Bacillus coagulans* 3,000 CFU/ ml	Dried ginger, *Zingiber officinale* powder 1–15 g/ kg diet	Indian carp, *Catla catla*	90 days	(54)
*Bacillus amyloliquefaciens* 10^9^ CFU/ g diet	*Spirulina platensis* 1 g/ kg diet	Nile tilapia, *Oreochromis niloticus*	60 days	(55)
*Lactobacillus plantarum* 10^8^ CFU/ kg diet	By-product from King oyster mushroom extract 5 g/ kg diet	White shrimp, *Litopenaeus vannamei*	8 weeks	(56)
*Bacillus* sp., *Pediococcus* sp., *Enterococcus* sp., and *Lactobacillus* sp. 1.34 × 10^10^ CFU/ kg diet	Pea 14% of diet	Senegalase sole, *Solea sengalensis*	73 days	(57)
*Pediococcus acidilactici* NA	Citrus flavonoids 0.003%	Rainbow trout, *Oncorhynchus mykiss*	63 days	(58)
*Bacillus subtilis* 10^7^ CFU/ kg diet	*Tetraselmis chuii* 100 g/ kg diet *Phaeodactylum tricornutum* 100 g/ kg diet	Gilthead seabream, *Sparus aurata*	4 weeks	(59)
*Bacillus coagulans* G1902 10^11^ CFU/ g diet	Oregano oil 1 ml/ kg diet	Turbot, *Scophthalmus maximus*	30 days	(60)
*Lactobacillus fermentum* *Lactobacillus plantarum* 10^8^ CFU/ g diet	Pea protein concentration 9%/ kg diet Rapeseed oil 19.8%/ kg diet	Atlantic salmon	38 days	(36)

Spices, which are widely available and inexpensive, can be very effective when combined with probiotics. For example, Ghafarifarsani et al. studied the effect of combined supplementation of Persian shallots and probiotics in the diet of *Danio rerio* (30). Their results showed that synergistic supplementation of Persian shallots and probiotics improved the growth performance of *Danio rerio* further compared to supplementation with shallot or probiotics alone (30). Another example of a spice tested in aquafeed is fenugreek. Bahi et al. investigated the effects of fenugreek seed administration, either alone or in combination with one of the following probiotics [*B. licheniformis* (TSB27), *L. plantarum or B. subtilis* (B46)], on growth performance parameters of gilthead sea bream (50). In their study, Bahi et al. reported that the combined effect of fenugreek and any probiotics significantly improved fish weight gain compared to fenugreek alone (50). However, in the study, Bahi et al. did not study the effect of probiotic as a single supplement to compare with the other treatments.

Superfoods also have great potential for use as dietary supplements and in combination with probiotics in aquafeeds. Like spirulina and the oyster mushroom, these two superfoods are widely available in the health food section, and often by-products are created when processed superfoods are produced commercially. Al-Deriny et al. investigated the effects of Spirulina and *Bacillus amyloliquefaciens* on growth performance and related parameters in Nile tilapia. Their results showed that administration of Spirulina and probiotics significantly improved growth in Nile tilapia compared to administration of control preparations and single preparations (Spirulina or probiotics) (55). A similar trend was also observed in white shrimp, where Prabawati et al. demonstrated that the sole administration of either royal oyster mushroom by-product extract or *L. plantarum* was not as great as the synergistic effect of both supplements (56).

Yu et al. reported that shrimp fed with a combination of medicinal herbs (consisting of woad, indigowoad root, weeping forsythia, bunge *Corydalis*, patchouli, and Mongolian milkvetch) and probiotics (*Bacillus spp*.) showed significantly better growth performance than shrimp fed a basal diet (21). Improved weight gain was also observed in olive flounder fed a combination of a medicinal herb mixture (*A. koreanum, G. uralensis Fischer, and P. ginseng*) and probiotic cocktail (52). In the study by Harikrishanan et al., it was reported that olive flounder fed only medicinal herb mixture without probiotic cocktail did not gain significant weight compared to the combined treatment (52). However, in both studies, Yu et al. and Harikrishanan et al. were unable to demonstrate the effect of a single administration of medicinal herbs or probiotics in their study.

Although many studies have reported positive results on the combination effect of medicinal herbs and probiotics, there are also reports of no significant effect of combining medicinal herbs and probiotics on the growth performance of aquaculture species. For example, one study showed that the use of 1% fermented cactus fruit liquid with probiotics improved the growth performance of red seabream but not at lower and higher level of supplement inclusion (28). Therefore, selecting an ideal combination and dose of medicinal herbs and probiotics is crucial for maintaining the health of aquaculture species and avoiding setbacks in aquaculture species growth performance.

## Combined effects of probiotics and medicinal herbs on the immune response and disease resistance of aquatic animals

Numerous studies have reported that probiotic mixtures in combination with medicinal herbs strengthen the immune system and improve disease resistance in aquaculture species, as shown in Tables 3, 4, respectively. Although the mechanism of action of the synergistic effects is not well-described, the improvement in immune response parameters is often used as a reference for the results. The positive effect of combined treatment, medicinal herbs, and probiotic can be observed in both healthy and infected fish in which these treatments reduce the oxidative stress level and restore the health of infected fish.

**Table 3:** Application of probiotic and medicinal herb combination in improving immune system of aquatic animals.

**Probiotics**	**Medicinal herbs**	**Aquaculture species**	**Duration**	**References**
*Bacillus*	*A. membranaceus, A. sinensis, and C. hupehensis*	Nile tilapia *Oreochromis niloticus*	56 days	(27)
*Lactobacillus fermentum* *Lactobacillus plantarum* 10^8^ CFU/ g diet	Pea protein concentration 9%/ kg diet Rapeseed oil 19.8%/ kg diet	Atlantic salmon	38 days	(36)
*Bacillus licheniformis* *Lactobacillus plantarum* *Bacillus subtilis* 20 g/ kg diet	Fenugreek seeds 50 g/ kg diet	Gilthead seabream, *Sparus aurata*	3 weeks	(50, 64)
*Bacillus coagulans* 3,000 CFU/ ml	*Mentha piperita* 2–8 g / kg diet	Indian carp, *Catla catla*	90 days	(53)
*Bacillus coagulans* 3,000 CFU/ ml	Dried ginger, *Zingiber offcinale* powder 1–15 g / kg diet	Indian carp, *Catla catla*	90 days	(54)
*Lactobacillus plantarum* 10^8^ CFU/ kg diet	By-product from King oyster mushroom extract 5 g/ kg diet	White shrimp, *Litopenaeus vannamei*	8 weeks	(56)
*Bacillus coagulans* G1902 10^11^ CFU/ g diet	Oregano oil 1 ml/ kg diet	Turbot, *Scophthalmus maximus*	30 days	(60)
*Lactobacillus plantarum* NA	Fermented lemon peel 0.1% of kg diet	Orange spotted grouper, *Epinephelus coioides*	8 weeks	(65)

**Table 4:** Application of probiotic and medicinal herb combination in stimulating disease resistance of aquatic animals.

**Probiotics**	**Medicinal herbs**	**Disease**	**Aquaculture species**	**Duration**	**References**
*Bacillus* sp., *Lactobacillus* sp., and *Yeast* sp.,	Korean herbs mix (undisclosed)	*Edwardsiella tarda*	Nile tilapia *Oreochromis niloticus*	28 days	(29)
*Lactobacillus sakei* BK19 1% of diet	Baical skullcap extract, *Scutellaria baicalensis* 1% of diet	*Edwardsiella tarda*	*Oplegnathus fasciatus* (Temminck and Schlegel)	6 weeks	(31)
*Lactobacillus plantarum, L. acidophilus, L. brevis, Bacillus subtilis*, and *Saccharomyces cerevisiae* Each 0.1% of diet	Combination of *Allium koreanum, Glycyrrhiza uralensis* and *Panax ginseng* 0.5 % of diet	*Streptococcus parauberis*	Olive flounder, *Paralichthys olivaceus*	12 weeks	(52)
*Lactobacillus plantarum* 10^8^ CFU/ kg diet	By-product from King oyster mushroom extract 5 g/ kg diet	Vibriosis due to *Vibrio* spp.	White shrimp, *Litopenaeus vannamei*	8 weeks	(56)
*Lactobacillus plantarum* NA	Fermented lemon peel 1–3% of diet	*Photobacterium damselae*	Orange spotted grouper, *Epinephelus coioides*	8 weeks	(65)
*Bacillus subtilis* 10^11^ CFU/ g	*Astragalus* polysaccharide 37% of diet, Tuckahoe polysaccharide 50% of diet	*Vibrio splendidus*	Sea cucumber, *Apostichopus japonicus*	4 weeks	(66)
*Pediococcus acidilactici* 0.2% of kg diet	Pistochio hulls derived polysaccharide 0.1% of kg diet	*Aeromonas hydrophila*	Nile tilapia, *Oreochromis niloticus*	56 days	(67)

Prabawati et al. suggested that a combination of king oyster mushroom (KOME) and probiotics *(Bacillus spp*.) could reduce the risk of infectious diseases caused by Vibrio in shrimp (56). In their report, synergistic treatment of both king oyster mushroom extract and probiotic outperformed single treatment supplement (KOME and probiotics) in many aspects of health. The single treatment (KOME or probiotic) increased the number of *L. bacillus* in the intestine but showed no significant difference from that of the control group. In contrast, the combined treatment with the herb and probiotic treatment drastically increased the number of *L. bacillus* bacteria in the gut of the shrimp. Although the synergistic treatment seemed to improve the number of *L. bacillus* significantly more than the control group, the effect was not obvious compared to the single-additive treatment (56). For the health status assessment, the disease resistance of shrimp against *V. alginolyticus* was improved in all treatments (combination and single treatment) compared to the shrimp in control. Shrimps in the combined treatment had significantly lower cumulative mortality due to the significant increase in immune responses, including phenoloxidase, respiratory burst, and lysozyme activity in shrimp as compared to the control and single supplement treatments (56).

Abarike et al. reported that the combined treatment of Chinese medicinal herbs and probiotics improves hemato-immunological, stress, and antioxidant parameters, and expression of HSP70 and HIF-1α mRNA to hypoxia, cold, and heat stress in Nile tilapia, in comparison with single supplement of Chinese herbs and probiotics (27). Harikrishnan et al. found that olive flounder infected with *S. parauberis* and treated with a combination of medicinal herbs (Korean rocky chive, Chinese liquorice ginseng) and a probiotic cocktail had significantly improved serum lysozymes, alternative complement activity, phagocyte activity, and oxidative burst activity compared to the uninfected control and *S. parauberis* infected fish treated with herbs alone (52). In a separate study, Harikrishanan et al. (31) investigated the protective effect of Baical skullcap herb and/or probiotic *Lactobacillus sakei* BK19 enriched diet on hematological and immunity status of rock bream against *Edwardsiella tarda*. When compared to the control and single supplement treatment (herb or probiotic), Harikrishanan et al. (31) found that treatment with Baical skullcap and *Lactobacillus sakei* BK19 effectively minimized mortality, restored altered hematological parameters, and enhanced innate immunity in rock bream against *E. tarda*. The combined treatment significantly improved the reactive oxygen species (ROS) and the reactive nitrogen species (RNS) in the infected fish already in the 1st week of treatment compared to the control and to the treatments with only one supplementation (31).

A study on gilthead sea bream showed that fenugreek seeds, either alone or in combination with one of the probiotics (*B. licheniformis, L. plantarum*, and *B. subtilis*), enhanced the humoral immune response of the fish (50). Another study with gilthead sea bream found that supplementing with probiotic combination of *B. licheniformis, L. plantarum*, and *B. subtilis* along with fenugreek increased hepatic superoxide dismutase and catalase, which are antioxidants that protect against oxidative stress (36).

The potential of the combination effect of probiotics and Tunisian date palm fruit was described by Estaban et al. In their study, the combined effect of both supplements was shown to increase mRNA expression of antioxidant enzymes in the mucosa of gilthead seabream (*Sparus aurata* L.) compared to treatments with a single supplement (68). However, the study lacked information on the control group. Guardiola et al. (69) investigated the combined effects of Tunisian date palm fruit and probiotics on serum antioxidant levels, humoral and cellular innate immune status, and mRNA expression of selected immune-related genes in the head-kidney and gut of European sea bass (*Dicentrarchus labrax*). In the study, Guardiola et al. indicated that the combination of supplements showed a better innate immune response in sea bass than treatments with single supplements (69).

However, not every combination of probiotics and medicinal herbs increases disease resistance in aquatic animals. For example, Villumsen et al. (58) showed that a combination of the probiotic *Pediococcus acidilactici* and the medicinal herb citrus flavonoids did not increase the resistance of rainbow trout (*Oncorhynchus mykiss*) to infection with *Yersinia ruckeri*. Therefore, further studies on the combination of probiotics and medicinal herbs for prophylactic purposes in aquaculture need to be conducted before the combination can be used in aquaculture health management.

## Conclusion and future perspectives

This review article revealed the promising findings of current probiotic and medicinal herb combinations as a feed additive for aquaculture uses. The innovative combinations of feed additives benefited the aquaculture industry by boosting the aquaculture species' growth performance, immune system, and disease resistance. In the future, research can be carried out to explore more possible combinations of feed additives adapted for relevant aquaculture species with bio-safety considerations. Existing knowledge gaps between findings from scientific research and practical use in aquaculture need to be filled before a new formulation of feed additive is feasible for commercial application. Therefore, a combination of multispecies of probiotic and medicinal herbs is an option for aquaculture species health management.

## Author contributions

KG: financial support. LW: project administration, writing—original draft, and writing—reviewing and editing. ZA: conceptualization and writing—reviewing and editing. WW: writing—reviewing and editing. NA: writing—original draft and reviewing and editing. HV: supervision and conceptualization. All authors contributed to the article and approved the submitted version.

## Funding

This work was funded by the Ministry of Education Malaysia under the scheme of Fundamental Research Grant Scheme (FRGS) with the code no. FRGS/1/2022/STG03/UMK/03/1 and Universiti Malaysia Kelantan Matching Grant (R/UMK MATCH/A0.700/00387A/008/2022). This research work was partially supported by Chiang Mai University.

## Conflict of interest

The authors declare that the research was conducted in the absence of any commercial or financial relationships that could be construed as a potential conflict of interest.

## Publisher's note

All claims expressed in this article are solely those of the authors and do not necessarily represent those of their affiliated organizations, or those of the publisher, the editors and the reviewers. Any product that may be evaluated in this article, or claim that may be made by its manufacturer, is not guaranteed or endorsed by the publisher.
